# Clinical and Immunohistochemical Features of Oral Angioleiomyoma: A Comprehensive Review of the Literature and Report of a Case in a Young Patient

**DOI:** 10.1155/2019/2498353

**Published:** 2019-02-27

**Authors:** Amerigo Giudice, Francesco Bennardo, Caterina Buffone, Ylenia Brancaccio, Francesca Maria Plutino, Leonzio Fortunato

**Affiliations:** ^1^School of Dentistry, Magna Graecia University of Catanzaro, Italy; ^2^Department of Health Sciences, Magna Graecia University of Catanzaro, Italy; ^3^Istituto Clinico De Blasi, Reggio Calabria, Italy

## Abstract

Angioleiomyoma (AL) is an uncommon benign soft tissue neoplasia arising from the tunica media of the smooth muscle cells. AL appears as a solitary and slow-growing mass and seldom is observed in oral tissues. We reported a rare case of AL involving the cheek of a 17-year-old young woman. A review of the English-language literature was performed entering the keywords “angioleiomyoma” and “oral” in the search fields of PubMed. 70 results were identified. Excluded were cases that were not in the oral cavity or not compatible with the AL diagnosis or report lacking immunohistochemical analysis. According to the exclusion criteria, we selected 30 studies that included 63 cases of AL. The results of the review showed an average age of 42.97 years with a prevalence between the fourth and fifth decade of life with a male-to-female ratio of 1.95 : 1. The most affected sites were palate, buccal mucosa of the cheek, lip, tongue, and gingiva. Surgical excision was the treatment of choice, and diagnosis was possible through histopathological and immunohistochemical analysis. SMA, vimentin, CD34, desmin, and S-100 were the most common markers to guide the histopathological diagnosis of oral AL. In conclusion, oral AL is a rare entity, especially in adolescence as in the reported case of AL of the cheek in a 17-year-old woman. The clinical aspects of AL did not allow clinicians to make a correct presumptive diagnosis. A scrupulous histopathological analysis and immunohistochemical examinations are fundamental to differentiate AL from other lesions.

## 1. Introduction

Leiomyoma is a circumscribed benign smooth muscle neoplasia that frequently occurs on the skin, especially on the lower extremities, in the gastrointestinal tract and female genital tract. Given the low presence of smooth muscle cells in the oral cavity, leiomyoma rarely localizes in the mouth: it develops from smooth muscle cells of tunica media or excretory ducts of salivary glands [1, 2].

In relation to the prevailing histological pattern, it can be classified into three types: solid leiomyoma, vascular leiomyoma (angioleiomyoma), and sporadic form of epithelioid leiomyoma (leioblastoma) [3].

Angioleiomyoma (AL) is the most common microscopic pattern affecting the oral cavity. Nevertheless, oral AL is a rare benign tumor. Typically, it occurs in middle-aged man between the fourth and sixth decades of life [4].

AL was found in the cheek, lips, tongue, mandible, palate, and gingiva. AL is commonly present as well circumscribed and slow-growing asymptomatic lesion [5–7].

The clinical aspects of AL are similar to many other lesions of the oral cavity. Therefore, the differential diagnosis of AL in the oral cavity includes other benign conditions of the salivary glands as mucocele, pleomorphic adenoma, mesenchymal tumors, lymphangioma, pyogenic granuloma, and schwannoma [8, 9].

The diagnosis is possible after histopathological and immunohistochemical analysis due to its nonspecific clinical features. Surgical excision is the treatment of choice of AL, and recurrences are very rare [9].

We report a rare case of AL in a 17-year-old woman and an extensive review of the literature on oral AL.

## 2. Case Report

A 17-year-old woman was referred to our Oral Pathology Unit by her family dentist to evaluate a solitary asymptomatic, mobile, and well-circumscribed mass in her left cheek.

The patient reported a swelling in the left cheek in the last two months. Her family and medical histories were irrelevant; she was not following any drug therapy; she did not smoke or usually consume alcohol.

Clinically, we observed a palpable hard-elastic mass, measuring 1.5 × 1.0 cm approximately, in the submucosal layer of the left cheek. The patient did not report pain; the skin and the covering mucosa were normal. An ultrasound scan (US) was prescribed and showed a hypoechoic homogenous mass with well-defined margins.

**Figure 1:**
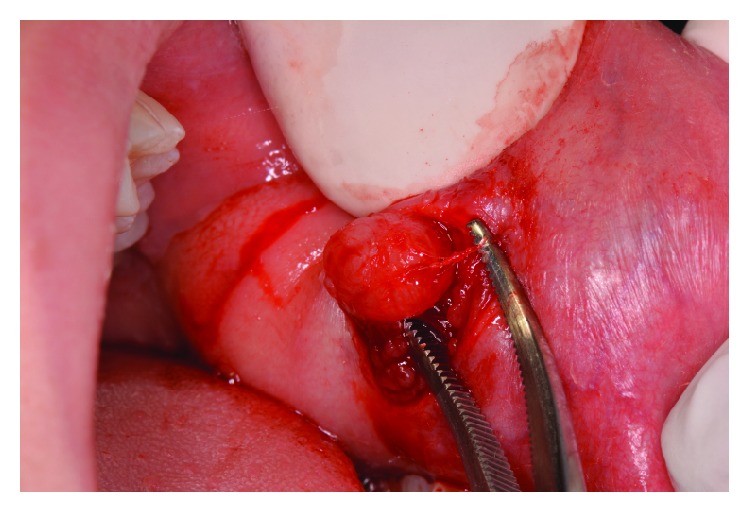
Surgical excision of the lesion.

The patient underwent an excisional biopsy under local anesthesia (Figure 1) after received an antibiotic prophylaxis therapy with 2 grams of amoxicillin 60 minutes before the surgery. She continued antibiotic therapy with 1 gram of amoxicillin every 12 hours until the 4^th^ postoperative day. As analgesic therapy, the patient received paracetamol 500 mg immediately after the surgery.

The specimen was stored in a tube containing formalin 10% and sent to a laboratory for histopathological analysis.

The tumor was well circumscribed with a thin fibrous capsule, and a sample obtained was firm, sharply circumscribed, yellow-white round to ovoidal nodule (1 × 0.6 cm in dimension). It was composed of uniform spindle smooth muscle cells with pale eosinophilic cytoplasm and blunt-ended or cigar-shaped nuclei, with slightly wavy contour, vesicular chromatin, and occasionally small nucleolus; in cross-sections, nuclei appeared surrounded by a clear halo as contained in boxes. Mitotic figures were very rarely seen (1/20 hpf). The cells were arranged in uniform interlacing bundles, with interposition of the low amount of fibrous connective tissue, and distributed around numerous small tortuous “slit-like” vessels, with virtual lumen and lined by normal-appearing endothelium but with no elastic lamina present, resembling a solid or capillary subtype appearance (closely compacted smooth muscle bundles), in contrast to venous (vessels have thick muscular walls that merge with smooth muscle bundles) and cavernous (dilated vascular channels with minimal smooth muscle that merges with smooth muscle bundles) subtypes; they have no clinical significance [1].

**Figure 2:**
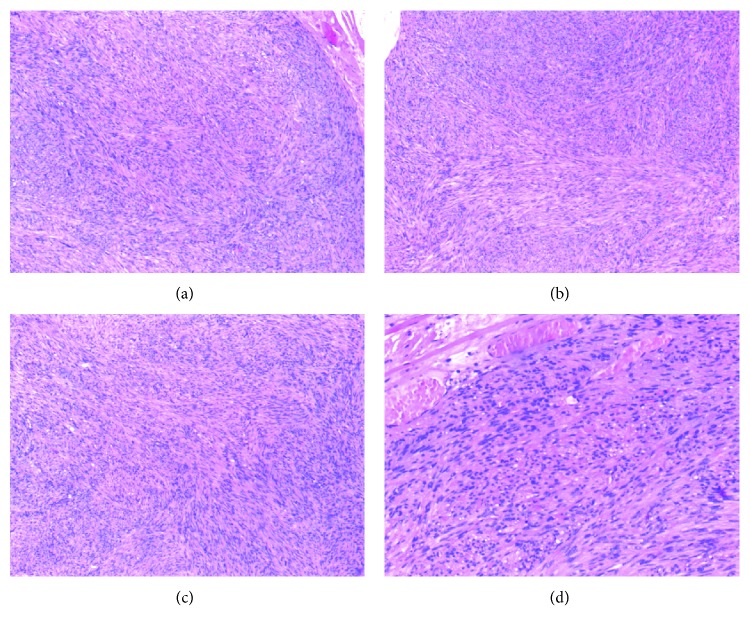
Histological findings of the case of angioleiomyoma reported. (a, b, c) The tumor is well circumscribed and shows an admixture of bundles of smooth muscle cells surrounding the blood vessels (4x); (d) AL: high-power view (10x).

Necrosis, atypical mitoses, and pleomorphism were not observed in the histological examinations (Figure 2).

**Figure 3:**
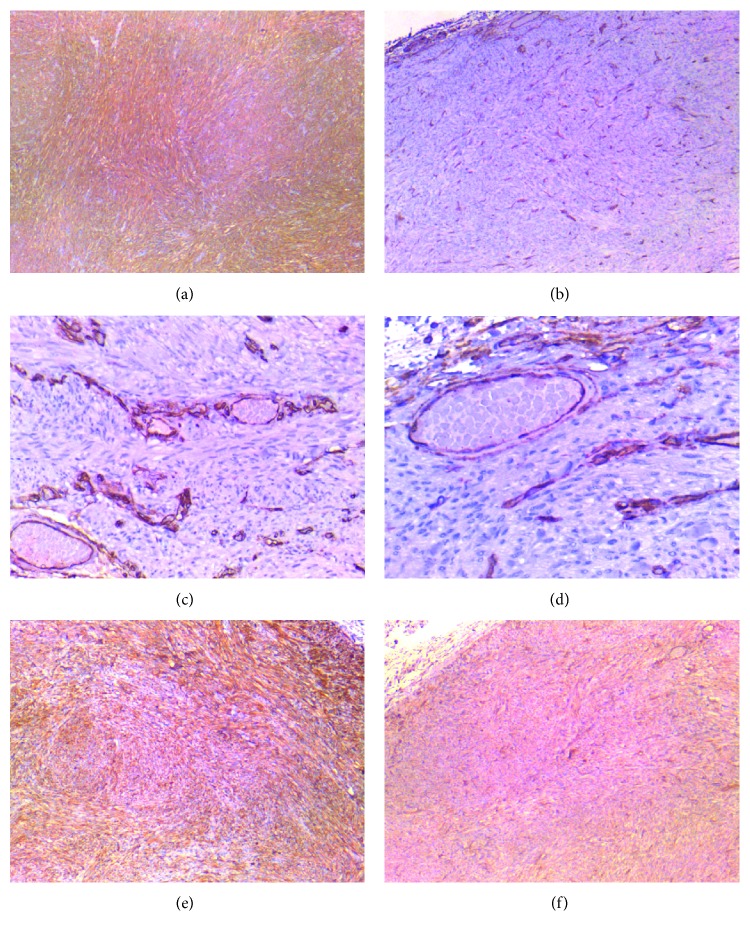
Immunohistochemical findings of the case of angioleiomyoma reported. (a) The tumor cells show strong and diffuse SMA expression (4x); (b) blood vessels endothelium marked with CD34 immunostaining (4x); (c) blood vessels endothelium marked with CD34 immunostaining at high magnification (10x); (d) blood vessels endothelium marked with CD34 immunostaining at high magnification (20x); (e) the tumor cells show strong and diffuse desmin expression (4x); (f) uniformly staining for vimentin (4x).

In addition to the histopathological analysis, immunohistochemical staining of the sample with *α*-smooth muscle actin (SMA), CD34, desmin, and vimentin was performed: the proliferating spindle cells were diffusely and strongly immunoreactive for SMA, desmin, and vimentin; the vascular spaces were consistently CD34-positive staining (Figure 3).

The histopathological and immunohistochemical analysis suggested the diagnosis of AL.

Follow-up examinations at 1 week, 4 weeks, and 6 months showed mucosal integrity and no sign of recurrence.

## 3. Review of the Literature

A review of the English-language literature was performed. The keywords “angioleiomyoma” and “oral” were entered in the search fields of PubMed. The research was conducted by considering the articles published until August 2018. 70 results were identified. Excluded were cases that were not in the oral cavity or not compatible with the AL diagnosis or report lacking immunohistochemical analysis.

According to the exclusion criteria, we selected 30 studies [3, 4, 8–34] that included 63 cases of AL. We analyzed patient's age, gender, tumor location, size, and immunohistochemical markers. The principal features and data pertaining the selected cases and those of the reported case are compiled in Table 1.

Age data were available in all selected studies, except for 14 cases described by Aitken-Saavedra et al. that reported only the mean age of 45.2 [34].

In our study, the average age was 42.97 years (range 2 months-79 years old) with a prevalence between the fourth and fifth decade of life.

**Figure 4:**
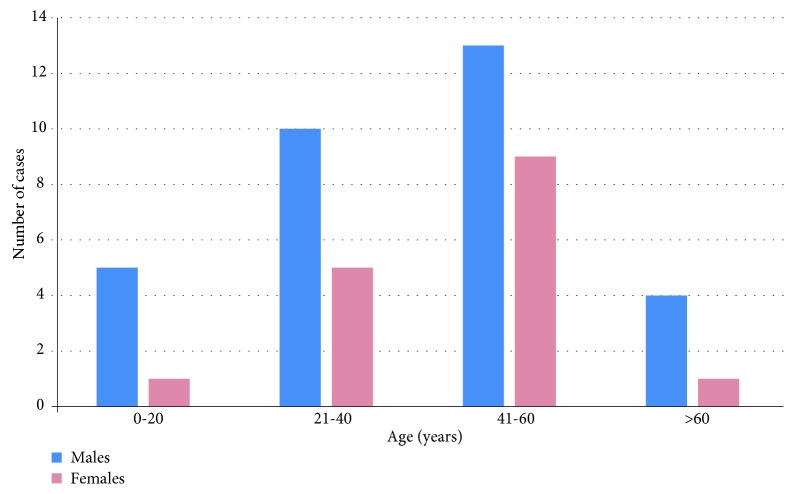
Distribution of age and sex in the 63 cases of angioleiomyoma of the oral cavity reviewed.

All studies included in our review, except one, reported the gender of the subjects. The analysis of the data collected suggested a male predilection with a male-to-female ratio of 1.95 : 1 (63 cases: 41 M, 21 F, and1 not reported). A graph of the age and gender distribution is reported in Figure 4.

In 63 cases reviewed, the analysis of localization in the oral cavity showed 19 in the lip (30%; 11 in the upper lip, 17.4%; 8 in the lower lip, 12.6%), 16 cases in the buccal mucosa of the cheek (or buccal space; 25.3%), 12 in the palate (19%), 5 in/on the tongue (7.9%), 5 in the gingiva (7.9%), and 3 in the mandible (4.7%). One lesion was observed in the retromolar area (1.58%). Other localizations were in the lower left back tooth region (1.58%) and in the lingual mucosa of the mandible (1.58%).

Size data were available in 46 of 63 cases. As reported in the literature, the size of the tumor can be very variable with a range from 0.5 × 0.5 to 3.5 × 3.3 × 2.0 cm.

The immunohistochemical analysis was performed for the differential diagnosis in all studies considered. This investigation revealed that the specimens were reactive to SMA (95.2%), desmin (73%), CD34 (44.4%), vimentin (42.8%), S-100 (7.9%), HHF-35 (7.9%), factor VIII (4.7%), h-caldesmon (1.58%), CD31 (1.58%), and NSE (1.58%).

Radiological investigations were not been prescribed in most cases considered; however, the investigations commonly described by many authors in the literature were MRI, CT, and US.

## 4. Discussion

Leiomyoma is described by the World Health Organization as a tumor of the soft tissue that arises from smooth muscle. It can be found in sites rich in smooth muscles such as the gastrointestinal tract, the myometrium, and the skin. Leiomyoma originating from smooth muscle cells of vessels lying on deep soft tissue is rare. Due to the lack of smooth muscle in the oral cavity, leiomyoma is relatively rare or uncommon. The possible sources of smooth muscle in the oral cavity include blood vessels, circumvallate papillae, and heterotopic smooth muscle [1, 2, 35].

In oral cavity, its origin is not well elucidated, but minor trauma, venous stasis, hormonal changes, and genetic translocation have been postulated as possible causes [36].

Leiomyoma is classified in three different types/major groups according to the prevailing histological pattern: the most common is the vascular form defined as AL (75%), followed by the solid form defined as leiomyoma (24%), and some cases of an epithelioid form defined as leiomyoblastoma are reported in the literature (<1%) [5, 37].

AL is a benign tumor resulting from the tunica media of smooth muscle cells of arterial and venous walls [37].

In the literature, it has been reported that AL accounts for 5% of all benign soft tumors and represents 3-3.9% of neoplasms that occur in the oral cavity [29, 36]. The incidence of AL in the oral cavity is rare and has been estimated to be roughly 0.065% [3].

Morimoto proposed a subclassification of AL describing solid, cavernous, and venous types [38]. On the authors' knowledge, only few authors have followed this subclassification reporting cases of intraoral AL: Liu et al. in a series of 21 tumors reported 5 solid, 6 venous, 9 cavernous, and 1 venous-cavernous AL [28]; Aitken-Saavedra et al. described 8 solid type, 4 cavernous type, and 2 venous type of AL [34].

Most of the AL are diagnosed between the fourth and sixth decade of life [38, 39], even if several studies have documented the tumor occurrence in subjects from 1 month to 84 years old [9]. There is only one study that reported a congenital tumor [21]. When AL occurs in this population, it is called leiomyomatous epulis, which clinically mimics a congenital granular cell tumor [28]. AL was rarely found during infancy and adolescence: only 6 reported cases of AL (5 males, 1 female) were diagnosed in patients younger than 20 years of age (Figure 4).

Conversely to extraoral localization, intraoral AL has a male predilection as reported in the literature by several authors [6, 24, 28, 34]. The results of our review of 63 cases confirm this with a 1.95 : 1 male-to-female ratio.

Many reports describe the lips as the most affected site with a frequency of 49%, followed by the palate, the buccal space, the mandible, the tongue, and the gingiva. Rare cases of intraosseous tumors are reported in the literature [25, 29]. The cheek was not the most frequent site of AL in the oral cavity, and the 16 cases reported in our review were observed in adult patients (age > 21). Therefore, we can consider our case rare of AL of the cheek reported in a young 17-year-old female teenager.

At clinical examination, AL appears in the oral cavity as a small, solitary, slowly developing mass [37], most commonly painless, and well localized. It presents as a palpable soft mass or elastic firm mass beneath the mucosa. The color of the mucous surface can be very variable, from normal to rosy or red [6].

Radiological investigations potentially useful for the diagnosis of AL are MRI, CT, and US. Yanagi et al., analyzing the usefulness of dynamic contrast-enhanced MRI in the differential diagnosis of AL in the buccal space, observed a very high signal intensity on T2WI and extremely high enhancement on Gd-T1WI; the inner aspect was homogeneous on T1WI and T2WI [40]. On CT images, AL appeared as a well-defined mass, heterogeneously well enhanced after the dye injection [22]. US revealed general hypoechogenicity with well-defined margins; in power Doppler mode, vascularity varies in density from low to high [41].

It is difficult to distinguish AL from the other solid lesions of the oral cavity, such as lymphangioma, hemangioma, fibroma, lipoma, pyogenic granuloma, and some other malignant lesions like angioleiomyosarcoma. AL is a benign tumor with a low rate of malignant transformation, and definitive diagnosis needs histopathological analysis. The presence of cellular atypia, pleomorphism, and necrosis at histological analysis is common in both AL and angioleiomyosarcoma, while the number of mitosis is the main criterion to establish the malignancy. Tumors that have 4-10 mitosis for 10 high-power fields (HPF) should be considered as potentially malignant, while those with at least 5 mitosis for 10 HPF as malignant [6, 19, 42].

The prognosis of AL also depends on surgical treatment, in fact the complete surgical excision represents the best strategy of treatment of AL [25].

In such cases, recurrence of AL occurs mainly due to incomplete excision of the lesion: in literature, recurrence have been reported in a few cases [19]. It is important to perform a complete tumor resection and a long-term follow-up observation [3].

Immunohistochemical analysis represents an essential tool in the diagnosis of AL. The most common markers assessed to confirm the diagnosis of AL are SMA, vimentin, CD34, desmin, and S-100 [28].

The results of this review reported a diffuse positivity to SMA in almost all cases. The markers vimentin, CD34, and desmin were reactive in about half of the cases. Only two study showed S-100 positivity in small nerve fibers in five cases of AL [24, 28]. Matiakis et al. found the positivity of h-caldesmon in one case: they identified this marker as more specific for smooth muscle fibers than SMA and desmin, also to differentiate AL from myopericytoma [7].

Maeda et al. showed that vascular walls, hardly identified by hematoxylin eosin stain, became visible through factor VIII immunohistochemical staining [10, 11]. The factor VIII is synthesized by endothelial cells, and its expression has been reported in numerous vascular neoplasms [43].

Endothelial cells also express CD31; therefore, antibodies to CD31 have been used as a tool to identify the vascular origin of neoplasms [44].

Kim et al., Gueiros et al., and Ishikawa et al. described a positive staining for HHF-35 that, in addition to reactivity for SMA, vimentin, desmin, and S-100, can assist in the diagnosis as an adjunct to H&E staining [22, 24, 29].

Cepeda et al. emphasized the importance of immunohistochemical analysis in order to differentiate AL from other types of spindle cell tumor, including leiomyoma (CD34^−^ and S-100^−^), myopericytoma (desmin^−^, CD34^−^, and S-100^−^), and myofibroma (desmin^−^, CD34^−^, and S-100^-/+^) [18].

Kim et al. and Aitken-Saavedra et al. reported a negativity of the sample for AE1/AE3 and CD68 antibodies and showed that only SMA can be elected as a good marker for AL and be of help in the diagnosis of this lesion [22, 34].

Gueiros et al. immunohistochemical analysis showed a negativity of the sample for D2-40 and a positive staining for SMA, desmin, CD34, HHF-35, and S-100 in 2 cases [24].

Each marker is a characteristic of a specific tissue, but its detection is useful only in combination with clinical judgement and the measurement of other markers.

In conclusion, oral AL is a rare entity, especially in adolescence. We have reported a new rare case of AL of the cheek in a young woman. The clinical aspects of AL did not allow us to make a correct presumptive diagnosis. A scrupulous histopathological analysis made it possible to identify the pathological entity of the lesion. Immunohistochemical examinations are fundamental to differentiate AL from other lesions. SMA, vimentin, CD34, desmin, and S-100 are the most commonly investigated markers to guide histopathological diagnosis of oral AL.

## Figures and Tables

**Table 1:** 

Study (year of publication)	*N* of cases	Age (years)	Gender	Tumor location	Size (cm)	Immunohistochemical markers
(1) Maeda et al. (1989) [10]	1	37	M	Upper lip	0.7 × 0.8	Vimentin+, desmin+, factor VIII+ S-100^−^, NSE+
(2) Maeda and Osaki (1989) [11]	1	49	F	Cheek	2.0 × 2.0	Vimentin+, desmin+, factor VIII+ S-100^−^
(3) Anastassov and Damme (1995) [12]	1	51	M	Upper lip	1.5 × 1.0	SMA+, factor VIII-
(4) Toida et al. (2000) [13]	1	10	M	Lower lip	2.0 × 1.5	SMA+, S-100^−^
(5) Marden et al. (2004) [14]	1	25	M	Tongue	3.2 × 3.0 × 1.2	SMA+, CD34^+^, CD31^+^
(6) Manor et al. (2007) [15]	1	39	M	Buccal mucosa	3.5 × 3.3 × 2.0	SMA+
(7) Scheper et al. (2007) [16]	1	67	M	Palate	5.0 × 5.0	SMA+
(8) Suresh et al. (2007) [17]	1	51	F	Mandible	1.7 × 1.5	SMA+, vimentin+, CD34^−^, S-100^−^
(9) Cepeda et al. (2008) [18]	5	39 27 43 36 48	F F F M M	Retromolar area Mandible Lower lip Upper lip Upper lip	0.9 × 0.6 × 1.0 1.4 × 1.3 × 1.0 0.7 × 0.5 × 0.4 1.5 × 1.0 × 1.0 1.7 × 1.0 × 1.0	SMA+, vimentin+, desmin+, CD34^−^, S-100^−^ SMA+, vimentin+, desmin+, CD34^−^, S-100^−^ SMA+, vimentin+, desmin+, CD34^−^, S-100^−^ SMA+, vimentin+, desmin+, CD34^−^, S-100^−^ SMA+, vimentin+, desmin+, CD34^−^, S-100^−^
(10) Keerthi et al. (2009) [19]	1	32	M	Cheek	4.5 × 4.0	SMA+
(11) Grossman et al. (2009) [20]	1	35	F	Palate	1.0 × 1.0 × 0.5	SMA+, vimentin+, desmin+, S-100^−^, HHF-35+, AE1/AE3-
(12) Kim et al. (2010) [21]	1	2 months	Not reported	Tongue	2.5 × 2.0	SMA+, vimentin-, desmin+, S-100^−^
(13) Kim et al. (2010) [22]	1	51	M	Buccal space	3.0 × 3.0	SMA+, CD34^−^, S-100^−^
(14) Nonaka et al. (2011) [23]	1	39	M	Tongue	2.0 ∅	SMA+
(15) Gueiros et al. (2011) [24]	3	54 66 54	M M M	Lower lip Upper lip Upper lip	1.0 × 1.0 Not reported 0.8 × 0.5 × 0.5	SMA+, desmin+, CD34^+^, S-100^+^, HHF-35+, D2-40- SMA+, desmin+, CD34^+^, S-100^−^, HHF-35+, D2-40- SMA+, desmin+, CD34^+^, S-100^+^, HHF-35+, D2-40-
(16) Patil et al. (2011) [25]	1	57	M	Lower left back tooth region	3.0 × 1.5 × 1.0	SMA+, vimentin+, desmin+, S-100^−^
(17) Menditti et al. (2012) [26]	1	14	M	Lingual mucosa of mandible	1.0/2.0 ∅	SMA+
(18) Eley et al. (2012) [27]	1	39	M	Palate	2.0 ∅	Desmin+, actin+
(19) Liu et al. (2014) [28]	14	62 49 36 51 49 10 30 20 60 34 58 18 19 47	F M F F F F F M M M M M M M	Buccal mucosa Buccal mucosa Buccal mucosa Buccal mucosa Palate Mandible Buccal mucosa Gingiva Palate Palate Lip Palate Tongue Buccal mucosa	1.5 ∅ 2.0 ∅ 2.0 ∅ 1.0 ∅ 1.0 ∅ 3.5 ∅ 3.0 ∅ 2.5 ∅ 1.5 ∅ 1.0 ∅ 6.0 ∅ 3.5 ∅ 3.5 ∅ 2.0 ∅	SMA+, vimentin+, desmin+, CD34^+^ in all cases S-100^+^ only in 3 cases (not specified)
(20) Tsuji et al. (2014) [3]	1	79	M	Palate	1.5 × 1.5	SMA+, desmin+
(21) Ishikawa et al. (2014) [29]	1	51	M	Tongue	1.1 ∅	SMA+, vimentin+, desmin+, CD34^−^, S-100^−^, HHF-35+
(22) Ranjan and Singh (2014) [30]	1	45	F	Gingiva	3.0 × 3.0	SMA+
(23) Inaba et al. (2015) [31]	1	45	F	Cheek	Not reported	SMA+, factor VIII+
(24) Osano et al. (2015) [9]	1	45	M	Cheek	2.0 ∅	SMA+, vimentin+, desmin+, CD34^−^, S-100^−^
(25) Arpağ et al. (2016) [32]	2	25 55	M F	Gingiva Gingiva	0.5 × 0.5 1.5 × 2.0	SMA+ SMA+
(26) Bajpai et al. (2016) [33]	1	39	M	Gingiva	3.0 × 3.0	SMA+, vimentin+, desmin+
(27) Hassona et al. (2017) [4]	1	52	F	Upper lip	Not reported	SMA+
(28) Rawal and Rawal (2017) [8]	1	70	M	Palate	2.0 × 1.5	SMA+
(29) Matiakis et al. (2018) [7]	1	51	M	Labial mucosa of the upper lip	0.8 ∅	SMA+, h-caldesmon+
(30) Aitken-Saavedra et al. (2018) [34]	14	Total mean 45.2	Male = 8 Female = 6	Lower lip Lower lip Lower lip Lower lip Upper lip Upper lip Upper lip Buccal mucosa Buccal mucosa Buccal mucosa Buccal mucosa Soft palate Soft palate Hard palate	Not reported Not reported Not reported Not reported Not reported Not reported Not reported Not reported Not reported Not reported Not reported Not reported Not reported Not reported	SMA+, AE1/AE3-, CD68^−^, desmin+, S-100^−^ in all cases CD34^+^ only in 10 cases (not specified)
Case report	1	17	F	Cheek	1.5 × 1.0	SMA+, vimentin+, desmin+, CD34^+^

## References

[1] Hachisuga T., Hashimoto H., Enjoji M. (1984). Angioleiomyoma. A clinicopathologic reappraisal of 562 cases. *Cancer*.

[2] Fletcher C. D. M., Unni K. K., Mertens F., WHO (2002). *Pathology and Genetics of Tumours of Soft Tissue and Bone*.

[3] Tsuji T., Satoh K., Nakano H., Kogo M. (2014). Clinical characteristics of angioleiomyoma of the hard palate: report of a case and an analysis of the reported cases. *Journal of Oral and Maxillofacial Surgery*.

[4] Hassona Y., Sawair F., Scully C. (2017). Angioleiomyoma of the upper lip. *BMJ Case Reports*.

[5] Barnes L., Eveson J. W., Reichart P., Sidransky D. (2005). *Pathology and Genetics of Head and Neck Tumours, World Health Organization Classification of Tumours*.

[6] Brooks J. K., Nikitakis N. G., Goodman N. J., Levy B. A. (2002). Clinicopathologic characterization of oral angioleiomyomas. *Oral Surgery, Oral Medicine, Oral Pathology, Oral Radiology, and Endodontology*.

[7] Matiakis A., Karakostas P., Pavlou A.-M., Anagnostou E., Poulopoulos A. (2018). Angioleiomyoma of the oral cavity: a case report and brief review of the literature. *Journal of the Korean Association of Oral and Maxillofacial Surgeons*.

[8] Rawal S. Y., Rawal Y. B. (2018). Angioleiomyoma (vascular leiomyoma) of the oral cavity. *Head and Neck Pathology*.

[9] Osano H., Ioka Y., Okamoto R. (2015). Angioleiomyoma of the cheek: a case report. *Journal of Oral Science*.

[10] Maeda Y., Hirota J., Osaki T., Hayashi K., Sonobe H., Otsuki Y. (1989). Angiomyoma of the upper lip: report of a case with electron microscopic and immunohistochemical observation. *British Journal of Oral and Maxillofacial Surgery*.

[11] Maeda Y., Osaki T. (1989). Angiomyoma of the cheek: a case report. *Journal of Oral and Maxillofacial Surgery*.

[12] Anastassov G. E., van Damme P. A. (1995). Angioleiomyoma of the upper lip: report of a case. *International Journal of Oral and Maxillofacial Surgery*.

[13] Toida M., Koizumi H., Shimokawa K. (2000). Painful angiomyoma of the oral cavity: report of a case and review of the literature. *Journal of Oral and Maxillofacial Surgery*.

[14] Marden F. A., Calilao G. C., Guzman G., Roy S. S. (2004). Glossal angiomyoma: imaging findings and endovascular treatment. *Head & Neck*.

[15] Manor E., Sion-Vardy N., Nash M., Bodner L. (2007). Angiomyoma of buccal vestibule: a rare case with a normal karyotype. *The Journal of Laryngology & Otology*.

[16] Scheper M. A., Nikitakis N. G., Meiller T. F. (2007). A stable swelling of the hard palate. *Oral Surgery, Oral Medicine, Oral Pathology, Oral Radiology, and Endodontology*.

[17] Suresh L., Matsumura E., Calixto L. E., Ruckert E., Aguirre A. (2007). Intraosseous angiomyoma of the mandible. *General Dentistry*.

[18] Gaitan Cepeda L. A., Quezada Rivera D., Tenorio Rocha F., Leyva Huerta E. R., Mendez Sanchez E. R. (2008). Vascular leiomyoma of the oral cavity. Clinical, histopathological and immunohistochemical characteristics. Presentation of five cases and review of the literature. *Medicina Oral Patologia Oral y Cirugia Bucal*.

[19] Keerthi R., Nanjappa M., Deora S. S., Kumaraswamy S. V. (2009). Angioleiomyoma of cheek: report of two cases. *Journal of Maxillofacial and Oral Surgery*.

[20] Grossmann S. d. M. C., Johann A. C. R., Castro W. H., Friedman H., Gomez R. S., Mesquita R. A. (2009). Anterior midline nodule of the hard palate. *Oral Surgery, Oral Medicine, Oral Pathology, Oral Radiology, and Endodontology*.

[21] Kim Y.-H., Jang Y.-W., Pai H., Kim S.-G. (2010). Congenital angiomyoma of the tongue: case report. *Dentomaxillofacial Radiology*.

[22] Kim H.-Y., Jung S.-N., Kwon H., Sohn W.-I., Moon S.-H. (2010). Angiomyoma in the buccal space. *Journal of Craniofacial Surgery*.

[23] Nonaka C. F. W., Pereira K. M. A., Miguel M. C. d. C. (2010). Oral vascular leiomyoma with extensive calcification areas. *Brazilian Journal of Otorhinolaryngology*.

[24] Gueiros L., Romanach M., Pires-Soubhia A., Pires F., Paes-De-Almeida O., Vargas P. (2009). Angioleiomyoma affecting the lips: report of 3 cases and review of the literature. *Medicina Oral Patología Oral y Cirugia Bucal*.

[25] Patil K., Mahima V., Srikanth H. (2011). Recurrent oral angioleiomyoma. *Contemporary Clinical Dentistry*.

[26] Menditti D., Laino L., Nastri L., Caruso U., Fiore P., Baldi A. (2012). Oral angioleiomyoma: a rare pathological entity. *In Vivo*.

[27] Eley K. A., Alroyayamina S., Golding S. J., Tiam R. N., Watt-Smith S. R. (2012). Angioleiomyoma of the hard palate: report of a case and review of the literature and magnetic resonance imaging findings of this rare entity. *Oral Surgery, Oral Medicine, Oral Pathology and Oral Radiology*.

[28] Liu Y., Li B., Li L., Liu Y., Wang C., Zha L. (2014). Angioleiomyomas in the head and neck: a retrospective clinical and immunohistochemical analysis. *Oncology Letters*.

[29] Ishikawa S., Fuyama S., Kobayashi T., Taira Y., Sugano A., Iino M. (2016). Angioleiomyoma of the tongue: a case report and review of the literature. *Odontology*.

[30] Ranjan S., Singh K. (2014). Gingival angioleiomyoma-infrequent lesion of oral cavity at a rare site. *Journal of Oral and Maxillofacial Pathology*.

[31] Inaba T., Adachi M., Yagisita H. (2015). A case of angioleiomyoma in the buccal space. *Odontology*.

[32] Arpağ O. F., Damlar I., Kılıç S., Altan A., Taş Z. A., Özgür T. (2016). Angioleiomyoma of the gingiva: a report of two cases. *Journal of the Korean Association of Oral and Maxillofacial Surgeons*.

[33] Bajpai M., Pardhe N., Kumar M. (2016). Angioleiomyoma of gingiva masquerading as pyogenic granuloma. *Journal of the College of Physicians and Surgeons Pakistan*.

[34] Aitken-Saavedra J., da Silva K. D., Gomes A. P. N. (2018). Clinicopathologic and immunohistochemical characterization of 14 cases of angioleiomyomas in oral cavity. *Medicina Oral Patología Oral y Cirugia Bucal*.

[35] Cherrick H. M., Dunlap C. L., King O. H. (1973). Leiomyomas of the oral cavity: review of the literature and clinicopathologic study of seven new cases. *Oral Surgery*.

[36] Ramesh P., Annapureddy S. R., Khan F., Sutaria P. D. (2004). Angioleiomyoma: a clinical, pathological and radiological review. *International Journal of Clinical Practice*.

[37] Enzinger F. M., Lattes R., Torloni H. (1969). *Histological Typing of Soft Tissue Tumours*.

[38] Morimoto N. (1974). Angioleiomyoma [vascular leiomyoma]-a clinicopathologic study. *Medical Journal of Kagoshima University*.

[39] Leung K.-W., Wong D. Y.-K., Li W.-Y. (1990). Oral leiomyoma: case report. *Journal of Oral and Maxillofacial Surgery*.

[40] Yanagi Y., Asaumi J.-I., Hisatomi M. (2003). Usefulness of dynamic contrast-enhanced MRI in the differential diagnosis of angioleiomyoma in the buccal space. *European Journal of Radiology Extra*.

[41] Gomez-Dermit V., Gallardo E., Landeras R., Echevarría F., Barredo R. G. (2006). Subcutaneous angioleiomyomas: gray-scale and color Doppler sonographic appearances. *Journal of Clinical Ultrasound*.

[42] Luaces Rey R., Lorenzo Franco F., Gómez Oliveira G., Patiño Seijas B., Guitián D., López-Cedrún Cembranos J. L. (2007). Oral leiomyoma in retromolar trigone. A case report. *Medicina Oral Patología Oral y Cirugia Bucal*.

[43] Little D., Said J. W., Siegel R. J., Fealy M., Fishbein M. C. (1986). Endothelial cell markers in vascular neoplasms: an immunohistochemical study comparing factor VIII-related antigen, blood group specific antigens, 6-keto-PGF1 alpha, and Ulex europaeus 1 lectin. *The Journal of Pathology*.

[44] Parums D. V., Cordell J. L., Micklem K., Heryet A. R., Gatter K. C., Mason D. Y. (1990). JC70: a new monoclonal antibody that detects vascular endothelium associated antigen on routinely processed tissue sections. *Journal of Clinical Pathology*.

